# Prevalence and risk factors of malnutrition in patients with pulmonary tuberculosis: a systematic review and meta-analysis

**DOI:** 10.3389/fmed.2023.1173619

**Published:** 2023-08-10

**Authors:** Ai Li, Su-yun Yuan, Quan-guo Li, Jin-xing Li, Xiang-yu Yin, Na-na Liu

**Affiliations:** Department of Respiratory Critical Care Medicine, The Second People's Hospital of Weifang, Weifang, Shandong, China

**Keywords:** pulmonary tuberculosis, malnutrition, prevalence, risk factors, systematic review, meta-analysis

## Abstract

**Background:**

Malnutrition is prevalent in patients with pulmonary tuberculosis (PTB) and is associated with a poor prognosis.

**Objective:**

This study aims to assess the prevalence and risk factors of malnutrition in patients with PTB.

**Methods:**

Studies related to the prevalence and risk factors of malnutrition in patients with PTB were searched through PubMed, Embase, Web of Science, and Cochrane Library databases from January 1990 to August 2022, and two researchers screened the literature, evaluated the quality, and extracted data independently. A random-effects model was used to pool the effect sizes and 95% confidence intervals. Subgroup analysis, meta-regression analysis, and sensitivity analysis were further performed to identify sources of heterogeneity and evaluate the stability of the results. Publication bias was assessed by Doi plot, Luis Furuya-Kanamori (LFK) asymmetry index, funnel plot, and Egger's tests.

**Results:**

A total of 53 studies involving 48, 598 participants were identified in this study. The prevalence of malnutrition was 48.0% (95% CI, 40.9–55.2%). Subgroup analysis revealed that malnutrition was more common among male gender (52.3%), bacterial positivity (55.9%), family size over 4 (54.5%), drug resistance (44.1%), residing in rural areas (51.2%), HIV infection (51.5%), Asian (51.5%), and African (54.5%) background. The prevalence of mild, moderate, and severe malnutrition was 21.4%, 14.0%, and 29.4%, respectively. Bacterial positivity (OR = 2.08, 95% CI 1.26–3.41), low income (OR = 1.44, 95% CI 1.11–1.86), and residing in rural areas (OR = 1.51, 95% CI 1.20–1.89) were risk factors of malnutrition in patients with PTB. However, male (OR = 1.04, 95% CI 0.85–1.26) and drinking (OR = 1.17, 95% CI 0.81–1.69) were not risk factors for malnutrition in patients with PTB. Due to the instability of sensitivity analysis, HIV infection, age, family size, smoking, and pulmonary cavity need to be reevaluated. Meta-regression suggested that sample size was a source of heterogeneity of prevalence. The Doi plot and LFK asymmetry index (LFK = 3.87) indicated the presence of publication bias for prevalence, and the funnel plot and Egger's test showed no publication bias for risk factors.

**Conclusion:**

This meta-analysis indicated that malnutrition was prevalent in patients with PTB, and bacterial positivity, low income, and those residing in rural areas were risk factors for malnutrition. Therefore, clinical workers should pay attention to screening the nutritional status of patients with PTB and identifying the risk factors to reduce the incidence of malnutrition and provide nutritional interventions early to improve the prognosis in patients with PTB.

## 1. Introduction

Tuberculosis is a chronic infectious disease caused by infection with *Mycobacterium tuberculosis* (1). Global Tuberculosis Report 2022 indicates that there will be ~10.6 million cases of tuberculosis worldwide, 6.40 million new cases of tuberculosis, and up to 1.60 million deaths in 2021, which makes it rank second as a cause of death from a single infectious agent after COVID-19 (2). It was also discovered that ~50% of patients with tuberculosis were diagnosed delayed during COVID-19, resulting in more severe symptoms in individuals who were hospitalized (3). Despite some progress made in the prevention and treatment of pulmonary tuberculosis (PTB) under the guidance of the World Health Organization, PTB remains widespread and is difficult to treat due to high transmission rates, rising drug resistance, lengthy treatment regimens, delayed access, and poor treatment compliance (4–6). Thus, the disease burden of tuberculosis remains severe.

As a chronic infectious disease of the respiratory system, PTB often presents with complications as the disease progresses, such as malnutrition, anemia, bronchiectasis, pulmonary hypertension, and respiratory failure (7–9). Malnutrition is currently widely studied in patients with PTB, but the prevalence of malnutrition varies widely between studies, ranging from 1.04 to 92.60% (10, 11). Malnutrition is not only an outcome of tuberculosis but also an important risk factor for the development of tuberculosis. According to a study, malnutrition may have a significant role in the development of tuberculosis. Patients with malnutrition may be more susceptible to developing the disease due to impaired innate and adaptive immunity to *Mycobacterium tuberculosis* (12). Additionally, patients with PTB frequently experience elevated energy needs, reduced appetite, low dietary intake, and malabsorption, all of which are closely associated with malnutrition (13). Patients with tuberculosis are frequently associated with high mortality, length of time to convert sputum, risk of drug resistance, and liver damage from anti-tuberculosis medications, which has a significant impact on the outcome and clinical prognosis (10, 14–16). Therefore, there is a positive impact on improving the treatment outcome and clinical prognosis by evaluating the nutritional status and its risk factors in patients with PTB.

A meta-analysis has reported the prevalence of malnutrition in patients with PTB (17), but the study is limited by small sample size, lack of subgroup analysis of demographic characteristics, and restricted to the Ethiopian region. Second, the awareness of risk factors of malnutrition remains controversial in patients with PTB. For instance, Kornfeld et al. (18) and Hussien et al. (19) suggested that male, HIV infection, and the pulmonary cavity were risk factors for malnutrition in patients with PTB, whereas Magassoub et al. (20) did not. Thus, we conduct this meta-analysis to assess the prevalence and risk factors of malnutrition in order to prevent and treat malnutrition early and improve the prognosis in patients with PTB.

## 2. Materials and methods

This study was reported according to the Preferred Reporting Items for Systematic Reviews and Meta-Analyses (PRISMA) guidelines (21) (see PRISMA checklist in Supplementary material). This protocol was registered in PROSPERO (No. CRD 42022358772).

### 2.1. Inclusion and exclusion criteria

Inclusion criteria were as follows: (1) study designs: including cross-sectional, cohort studies, and case-control studies; (2) participants: patients with PTB; (3) articles with diagnostic criteria of PTB (including bacteriology as well as clinical diagnosis) and malnutrition [including Body Mass Index (BMI), Nutritional Risk Screening 2002 (NRS-2002), Patient-Generated Subjective Global Assessment (PG-SGA), Subjective Global Assessment (SGA), Mini Nutritional Assessment (MNA), Short-Form Mini-Nutritional Assessment (MNA-SF), Global Leadership Initiative on Malnutrition (GLIM) criteria, European Society for Clinical Nutrition and Metabolism (ESPEN) criteria, French criteria 2007, Malnutrition Screening Tool (MST), Malnutrition Universal Screening Tool (MUST), and Geriatric Nutritional Risk Index (GNRI)]; (4) outcomes: prevalence and risk factors of malnutrition; and (5) articles published in English.

Exclusion criteria were as follows: (1) duplicate studies, reviews, and animal studies; (2) relevant outcomes not available; (3) full text not available, conference abstract; and (4) studies with extrapulmonary tuberculosis.

### 2.2. Search strategy

Studies related to the prevalence and risk factors for malnutrition in patients with PTB published between January 1990 and August 2022 were searched through PubMed, Embase, Web of Science, and the Cochrane Library databases. We used a combination of Medical Subject Headings terms and free terms for the search. The detailed search strategy is shown in Supplementary Table 1.

### 2.3. Study selection, data extraction, and quality assessment

Two researchers (AL and S-YY) screened the literature and extracted relevant data independently, and any disagreements were resolved through discussion, and if unresolved, a final decision was made by a third person (N-NL). The information extracted from the studies included the study's first author, publication year, country, study design, sample size, the number of patients with malnutrition, the definition of malnutrition, and risk factors. The quality of cross-sectional studies was evaluated using the Agency for Healthcare Research and Quality with 0 to 3 being as low quality, 4 to 7 as moderate quality, and 8 to 11 as high quality. The Newcastle-Ottawa Scale (22) was used to assess the quality of cohort studies and case-control studies, with 0–3 being as low quality, 4–6 as moderate quality, and ≥7 as high quality.

### 2.4. Statistical analysis

Prevalence and risk factors were pooled when at least three and more studies reported the same outcome, and with the consideration of heterogeneity between studies, we used the random effects model to calculate the effect sizes of prevalence and risk factors with their 95% confidence intervals (95% CI) (23). Heterogeneity was assessed using Chi-square and I^2^ tests, and sources of heterogeneity were explored by subgroup analysis (including gender, malnutrition degree, age, bacteriology, family size, drug resistance, residence, HIV infection, and region) as well as meta-regression. Sensitivity analysis was conducted by the leave-one-out method. As the conventional funnel plot was not applicable for assessing publication bias of prevalence (24, 25), we used the Doi plot and the Luis Furuya-Kanamori (LFK) asymmetry index to assess publication bias (26), with LFK index within ±1, between ±1 and ±2, and above ±2 being considered as no asymmetry, minor asymmetry, and major asymmetry, respectively. Publication bias was tested by funnel plot and Egger's test when risk factors were pooled with more than 10 studies. *P*-values of < 0.05 was considered statistically significant. Meta-analyses were performed by using Stata 14.0 software.

## 3. Results

### 3.1. Literature selection process

**Figure 1:**
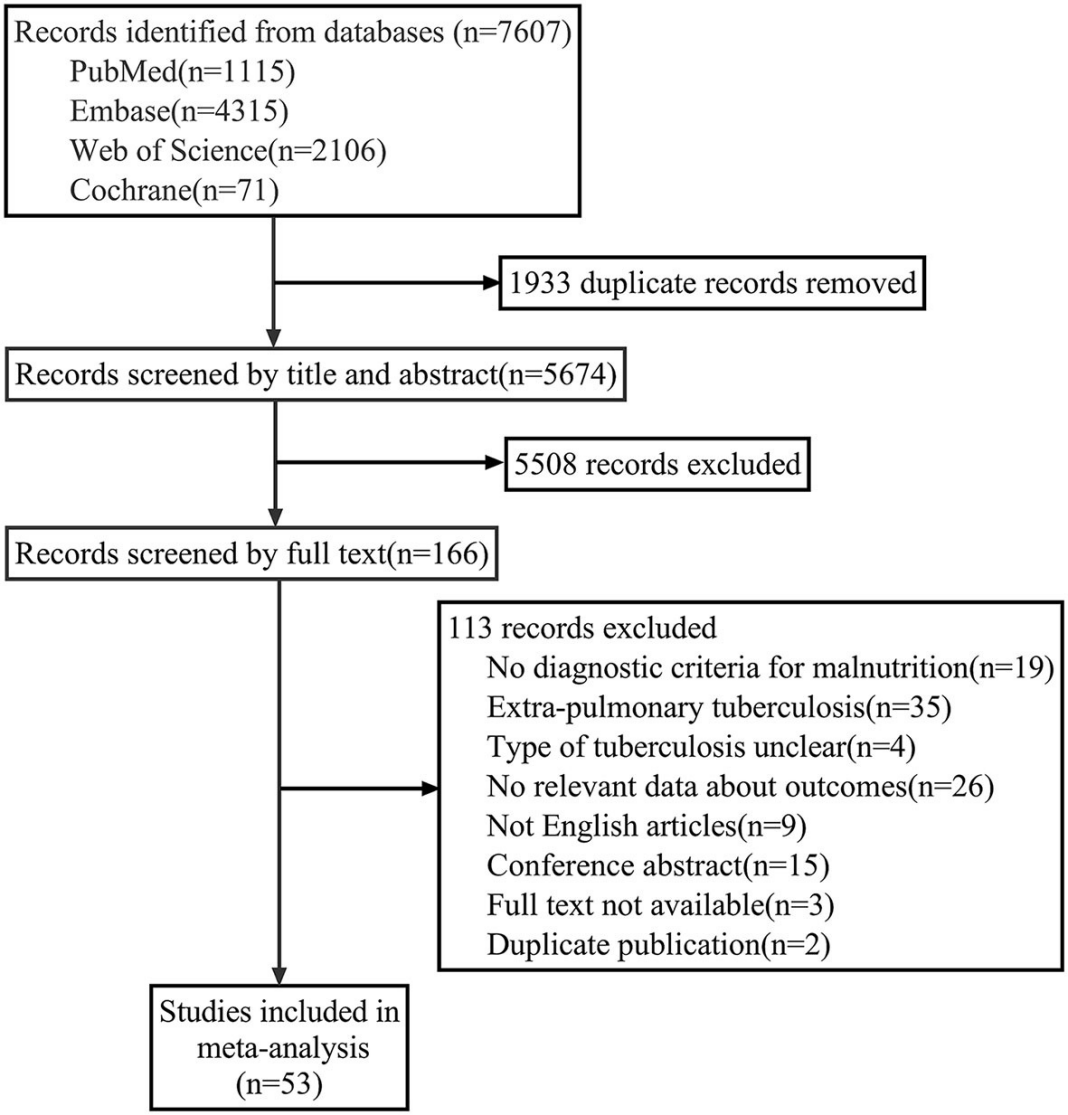
Flow diagram of literature selection.

A total of 7, 607 publications were obtained from the initial search, and 53 studies were enrolled for meta-analysis after selecting according to inclusion and exclusion criteria. The flow diagram of the literature selection is shown in Figure 1.

### 3.2. Basic characteristics and quality assessment of the included studies

These studies included 34 cross-sectional studies and 19 cohort studies with a total of 48, 598 patients with PTB; 26 studies were from Asia, 20 from Africa, 6 from the Americas, and 1 from Europe. The prevalence of malnutrition was reported in 53 research studies, while risk factors for malnutrition (such as gender, age, smoking, HIV infection, and income level) have been evaluated in 27 studies. A total of 51 studies used the BMI, one used NRS-2002, and one used PG-SGA for assessing malnutrition. Table 1 presents the basic characteristics of the incorporated studies, and the quality assessment is shown in Supplementary Tables 2, 3.

**Table 1:** Characteristics of the included studies.

**Study's first author**	**Publication year**	**Country**	**Study design**	**Simple size**	**Number of cases**	**Age**	**Definition of malnutrition**	**Risk factors**	**Study quality**
Baluku et al. et al. (27)	2022	Uganda	Cross-sectional	354	186	≥15	BMI < 18.5 kg/m^2^	-	8
Soeroto et al. (28)	2022	Indonesia	Cohort	315	199	≥18	BMI < 18.5 kg/m^2^	Gender; age; smoking; cavity	7
Iqbal et al. (29)	2022	Pakistan	Cross-sectional	252	169	31.97 ± 15.34	BMI < 18.5 kg/m^2^	-	8
Tao et al. (30)	2021	China	Cross-sectional	9,970	2,391	49.8 ± 19.7	BMI < 18.5 kg/m^2^	-	7
Magassouba et al. (20)	2021	Guinea	Cohort	218	141	≥18	BMI < 18.5 kg/m^2^	Gender; residence; HIV; cavity	6
Li et al. (31)	2021	China	Cross-sectional	246	149	≥18 (24–65)	NRS-2002≥3	-	6
Asemahagn (16)	2021	Ethiopia	Cohort	282	198	≥15	BMI < 18.5 kg/m^2^	-	5
Sahile et al. (32)	2021	Ethiopia	Cohort	330	159	≥18	BMI < 18.5 kg/m^2^	-	7
Singla et al. (33)	2021	India	Cohort	250	207	-	BMI < 16 kg/m^2^ (severe)	-	5
Hussien and Ameni (19)	2021	Ethiopia	Cross-sectional	450	232	Apr-81	BMI < 18.5 kg/m^2^	HIV; smoking; drinking; family size	7
Baluku et al. (34)	2021	Uganda	Cohort	473	276	39.0 ± 14.0	BMI < 18.5 kg/m^2^	-	6
Song et al. (35)	2021	China	Cross-sectional	8,957	2,121	All	BMI < 18.5 kg/m^2^	Gender; age; HIV; smoking; drinking	8
Kassa et al. (36)	2021	Ethiopia	Cross-sectional	515	388	31.88 ± 12.18	BMI < 18.5 kg/m^2^	Bacteriology	6
Montes et al. (10)	2021	Guatemala	Cross-sectional	3,945	41	≥18	BMI < 18.5 kg/m^2^	-	7
Kitonsa et al. (37)	2020	Uganda	Cross-sectional	491	118	≥15	BMI < 18.5 kg/m^2^	Bacteriology	6
Shimouchi et al. (38)	2020	Japan	Cross-sectional	192	69	-	BMI < 18.5 kg/m^2^	Bacteriology	7
Musuenge et al. (39)	2020	Saharan	Cross-sectional	302	108	≥15	BMI < 18.5 kg/m^2^	Age; Gender; HIV; family size smoking; drinking; low income; residence	6
Ko et al. (40)	2020	Korea	Cross-sectional	215	44	≥20	BMI < 18.5 kg/m^2^	Bacteriology	6
Kornfeld et al. (18)	2020	India	Cohort	389	184	25–60	BMI < 18.5 kg/m^2^	Gender; low income; smoking; drinking; cavity	6
White et al. (9)	2020	Philippines	Cross-sectional	634	232	≥18	BMI < 18.5 kg/m^2^	Residence	6
Campos et al. (41)	2019	Mexico	Cross-sectional	39	6	≥15	BMI < 18.5 kg/m^2^	-	4
Kubiak et al. (42)	2019	India	Cross-sectional	919	564	≥6	BMI < 18.5 kg/m^2^	-	7
Rashak et al. (43)	2019	Mexico	Cross-sectional	4,954	724	≥18	BMI < 18.5 kg/m^2^	-	6
Hoyt et al. (44)	2019	India	Cross-sectional	173	100	≥15	BMI < 18.5 kg/m^2^	Gender; smoking; drinking; cavity	6
Hussien et al. (45)	2019	Ethiopia	Cross-sectional	372	235	31.55 ± 15.79	BMI z-score < −2 (age < 18);BMI < 18.5 kg/m^2^ (age≥18)	Gender; HIV; smoking; drinking; family size; residence; cavity	5
Feleke and Feleke (46)	2019	Ethiopia	Cross-sectional	1,297	735	27.78 ± 13.98	BMI < 18.5 kg/m^2^	-	7
Lazzari et al. (47)	2018	Brazil	Cross-sectional	108	39	≥18	BMI < 18.5 kg/m^2^	-	5
Patsche et al. (48)	2017	Guinea	Cross-sectional	141	71	≥15	BMI < 18.5 kg/m^2^	-	7
Hochberg et al. (49)	2017	India	Cross-sectional	405	244	14–81	BMI < 18.5 kg/m^2^	Gender	6
Yen et al. (15)	2017	China	Cohort	2,226	577	≥18	BMI < 18.5 kg/m^2^	-	7
Park et al. (50)	2016	Korea	Cohort	218	53	41.7 ± 14.2	BMI < 18.5 kg/m^2^	Gender; bacteriology	8
Chung-Delgado et al. (51)	2014	Peru	Cohort	201	53	33.6 ± 16.2	BMI < 18.5 kg/m^2^	-	6
Putri et al. (52)	2014	Indonesia	Cohort	212	152	37.5 ± 11.9	BMI < 18.5 kg/m^2^	Gender	7
Bhargava et al. (53)	2013	India	Cohort	1,523	1,358	≥18	BMI < 18.5 kg/m^2^	Gender	7
Mupere et al. (54)	2012	Uganda	Cohort	747	310	≥18	BMI < 18.5 kg/m^2^	Gender; HIV; smoking; drinking	6
Podewils et al. (55)	2011	Latvia	Cohort	995	199	≥18	BMI < 18.5 kg/m^2^	Gender; cavity; drinking	8
Kawai et al. (56)	2011	Tanzania	Cohort	887	282	18–65	BMI < 18.5 kg/m^2^	Gender; HIV	6
Lawson et al. (57)	2008	Nigeria	Cross-sectional	625	179	≥15	BMI < 18.5 kg/m^2^	HIV	7
Lettow et al. (58)	2004	Malawi	Cross-sectional	779	456	18–59	BMI < 18.5 kg/m^2^	HIV	6
Madebo et al. (59)	1997	Ethiopia	Cross-sectional	239	187	≥15	BMI < 18.5 kg/m^2^	-	7
Muchsin et al. (60)	2019	Indonesia	Cross-sectional	116	64	≥18	BMI < 18.5 kg/m^2^	-	3
Kennedy et al. (61)	1996	Tanzania	Cohort	148	106	≥18	BMI < 18.5 kg/m^2^	Gender	5
Swaminathan et al. (62)	2008	India	Cross-sectional	174	82	31.1 ± 7.5	BMI < 18.5 kg/m^2^	Gender	7
Nandasena et al. (63)	2019	Sri Lanka	Cohort	424	295	All	BMI < 18.5 kg/m^2^	-	6
Frediani et al. (64)	2016	Georgia	Cohort	191	46	≥18	BMI < 18.5 kg/m^2^	-	5
Sari et al. (65)	2019	Indonesia	Cross-sectional	39	13	≥16	BMI < 18.5 kg/m^2^	-	6
Abdus Salam (11)	2018	India	Cross-sectional	54	50	20–50	BMI < 18.5 kg/m^2^	Bacteriology	4
PrayGod et al. (66)	2011	Tanzania	Cross-sectional	355	203	≥15	BMI < 18.5 kg/m^2^	Gender	6
Cegielski et al. (67)	2013	Georgia	Cohort	439	233	≥18	BMI < 18.5 kg/m^2^	-	5
Piva et al. (68)	2013	Brazi	Cross-sectional	34	13	15–59	BMI < 18.5 kg/m^2^	-	6
Dodor (69)	2008	Ghana	Cross-sectional	570	291	≥18	BMI < 18.5 kg/m^2^	-	7
Lin et al. (70)	2021	China	Cross-sectional	117	59	70.7	PG-SGA	Gender; family size; low income; cavity	6
Pakasi et al. (71)	2009	Indonesia	Cross-sectional	121	103	18–55	BMI < 18.5 kg/m^2^	-	4

### 3.3. The overall prevalence of malnutrition in patients with PTB

**Figure 2:**
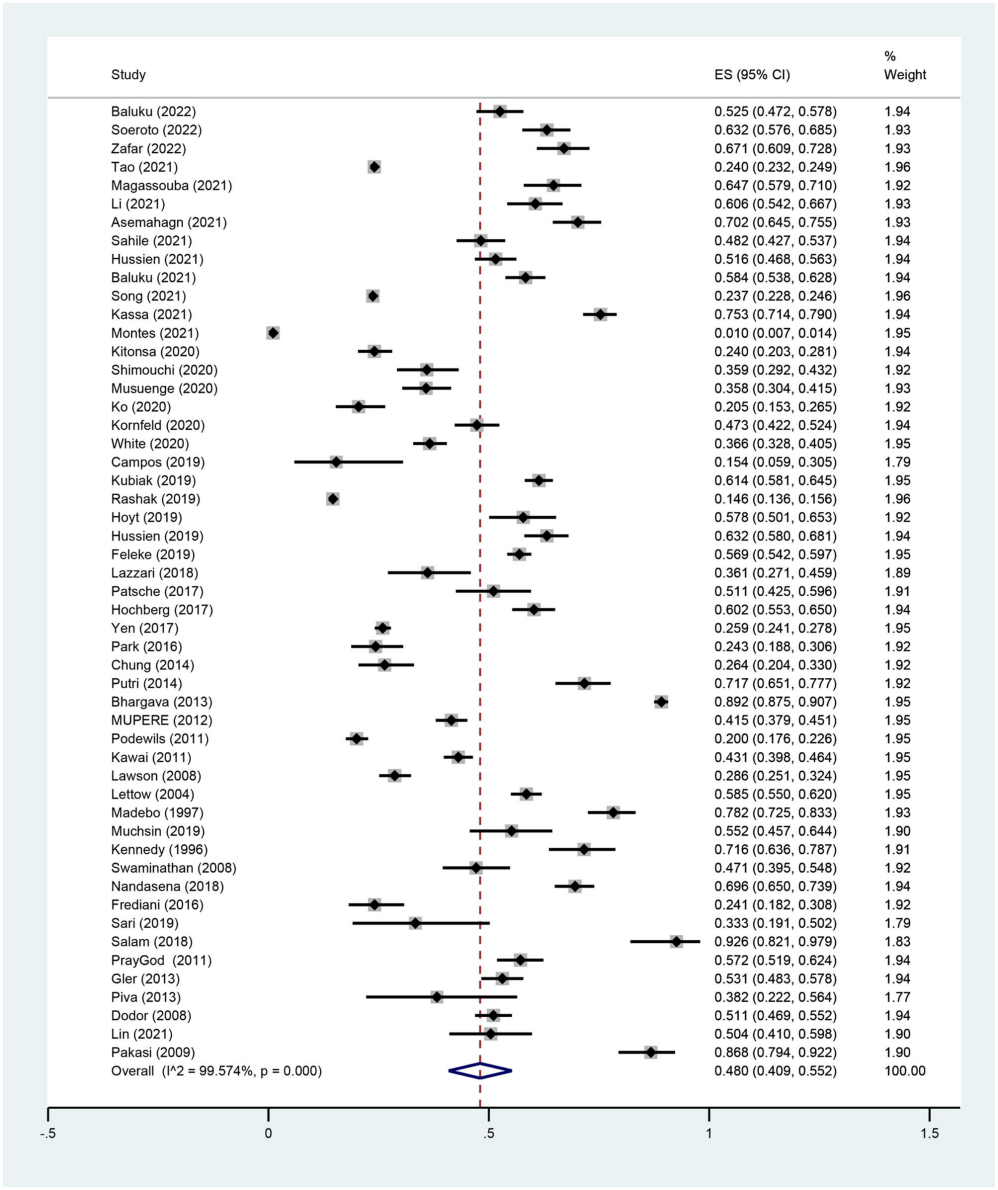
Forest plot of the prevalence for malnutrition in patients with PTB.

In total, 53 studies described the prevalence of malnutrition among PTB patients. Since Singla et al. (33) described only severe malnutrition, this study was not included to avoid any impact on the overall prevalence. The results showed that the overall prevalence of malnutrition was 48.0% (95% CI, 40.9–55.2%, *P* < 0.001) with significant heterogeneity between studies (I^2^ = 99.6%, *P* < 0.001) (Figure 2).

### 3.4. Subgroup analysis of the prevalence of malnutrition in patients with PTB

The results of the subgroup analysis were as follows: the prevalence of malnutrition was slightly higher in male subjects than in female subjects (52.3 vs. 50.8%). The prevalence of mild, moderate, and severe malnutrition was 21.6, 14.0, and 29.4%, respectively. The prevalence of malnutrition was 49.6 and 33.4% among those aged < 65 and ≥65 years. The prevalence of malnutrition was higher among PTB patients with bacterial positivity, family size over 4, drug resistance, residing in rural areas, and HIV infection. The prevalence of malnutrition was significantly higher among PTB patients in Africa and Asia compared with the Americas, and it is not described in Europe as only one study was offered. The summary results of the subgroup analysis are shown in Table 2.

**Table 2:** Subgroup analysis of the prevalence of malnutrition in pulmonary tuberculosis.

**Subgroups**	**No. of studies**	**Prevalence (95%*CI*)**	**Heterogeneity**	
			***I^2^***	***P*-value**
Gender				
Male	18	52.3% (39.6%, 64.9%)	99.3%	< 0.001
Female	18	50.8% (37.0%, 64.6%)	98.6%	< 0.001
Malnutrition degree				
Mild	10	21.6% (18.3%, 25.0%)	86.4%	< 0.001
Moderate	10	14.0% (11.7%, 16.5%)	79.8%	< 0.001
Severe	16	29.4% (19.2%, 40.9%)	98.9%	< 0.001
Age				
≥65	3	33.4% (19.7%, 48.5%)	59.0%	< 0.001
< 65	8	49.6% (33.4%, 65.8%)	99.3%	< 0.001
Bacteriology				
Positive	27	55.9% (47.9%, 63.8%)	99.1%	< 0.001
Negative	8	40.0% (21.8%, 59.8%)	96.7%	< 0.001
Family size				
≤ 4	4	48.8% (41.8%, 55.8%)	78.5%	< 0.001
>4	4	54.5% (39.7%, 68.9%)	93.9%	< 0.001
Drug resistance				
Yes	14	44.1% (32.3%, 56.3%)	99.0%	< 0.001
No	4	20.5% (6.9%, 39.1%)	99.9%	< 0.001
Residence				
Rural	3	51.2% (29.5%, 72.6%)	95.2%	< 0.001
Urban	4	41.1% (32.1%, 50.4%)	91.9%	< 0.001
HIV infection				
Yes	10	51.5% (42.9%, 60.1%)	90.8%	< 0.001
No	9	47.7% (39.1%, 56.2%)	95.4%	< 0.001
Region				
Asia	25	51.5% (42.2%, 60.7%)	99.5%	< 0.001
Africa	20	54.1% (47.7%, 60.4%)	97.4%	< 0.001
America	6	19.0% (7.2%, 34.4%)	99.4%	< 0.001

### 3.5. Meta-regression

To further explore the sources of heterogeneity, a multivariate meta-regression analysis was performed based on the publication year, sample size, study design, and study quality, and the results showed that sample size was a possible source of heterogeneity (I^2^ = 93.58%, adjusted R^2^ = 36.30%, *t* = −2.05, *P* = 0.046) (Table 3). The studies were divided into two groups according to the median sample size (*n* = 342) and the results showed that both the small sample studies (I^2^ = 96.7%, *P* < 0.001) and the large sample studies (I^2^ = 99.8%, *P* < 0.001) did not reduce heterogeneity.

**Table 3:** Multivariate meta-regression analysis of the prevalence for malnutrition in patients with PTB.

**Covariates**	**Coef**.	**Std. err**.	**t**	***P***	**95% Conf. interval**	
Publication year	−0.005	0.004	−1.16	0.254	−0.013	0.004
Simple size	−0.265^a^	0.129^a^	−2.05	0.046	−0.526^a^	−0.004^a^
Study design						
Cross–sectional study	−0.009	0.051	−0.17	0.866	−0.112	0.095
Study quality	−0.014	0.027	−0.52	0.605	−0.068	0.040
Region						
Africa	0.295	0.174	1.69	0.098	−0.056	0.645
Asia	0.298	0.176	1.70	0.097	−0.056	0.653
America	−0.003	0.193	−0.01	0.989	−0.392	0.386

^a^Original data × 10000.

### 3.6. Sensitivity analysis

**Figure 3:**
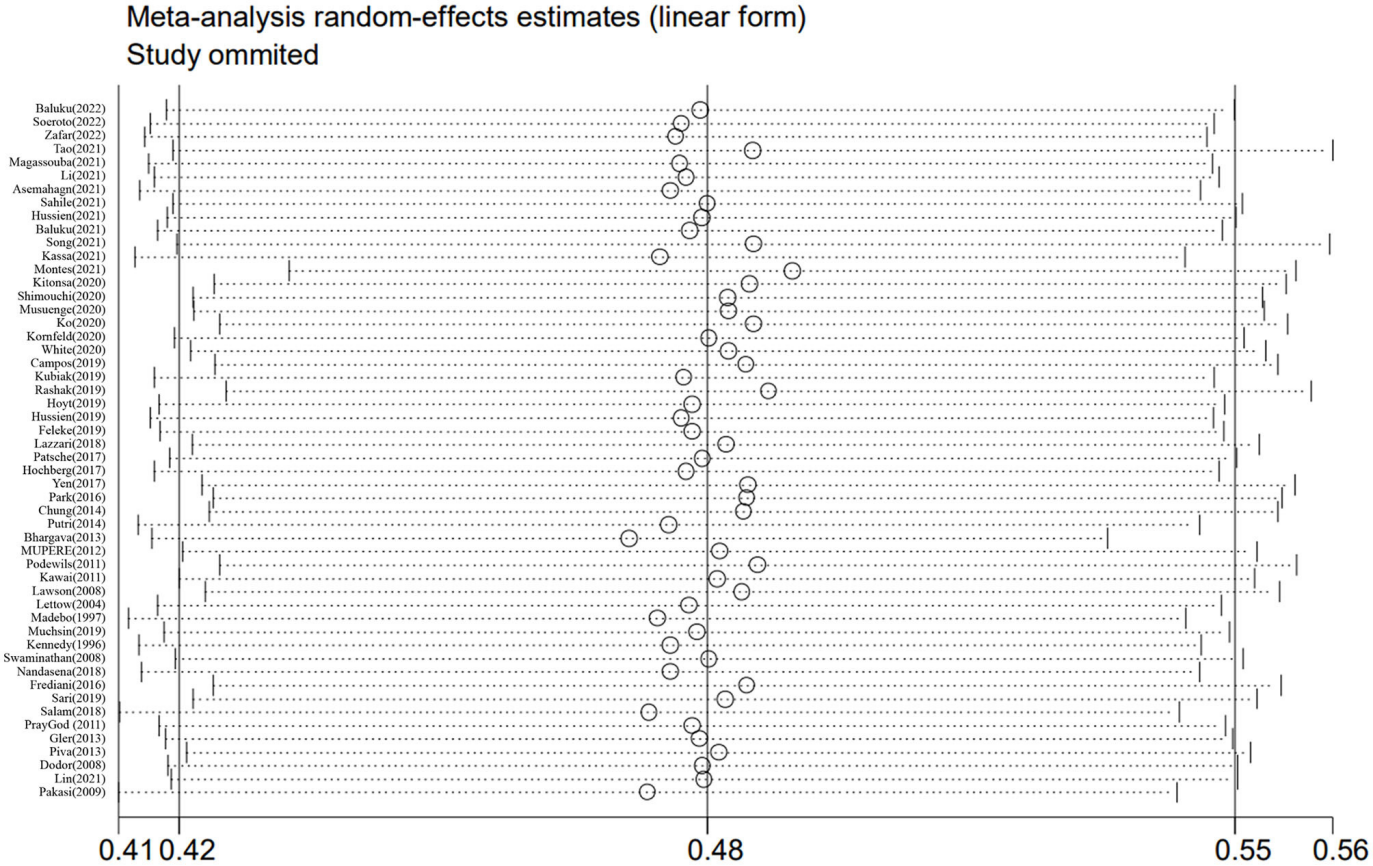
Sensitivity analysis of the prevalence of malnutrition in patients with PTB.

Sensitivity analysis was performed by excluding individual studies one by one, and the results showed that the prevalence of malnutrition ranged from 47.1 to 49.4%, with little difference from the overall prevalence, which suggested that this meta-analysis was stable and reliable (Figure 3).

### 3.7. Risk factors for malnutrition in patients with PTB

#### 3.7.1. Gender

A total of 18 studies reported the association between male and malnutrition in patients with PTB. The meta-analysis showed that male was not a risk factor for malnutrition (OR = 1.04, 95% CI 0.85–1.26, *P* = 0.720), and there was significant heterogeneity between studies (I^2^ = 75.4%, *P* < 0.001). The meta-regression analysis showed that publication year (β_coef_=-0.008, *P* = 0.681), sample size (β_coef_=-0.000, *P* = 0.963), study design (β_coef_=0.236, *P* = 0.314), and study quality (β_coef_=-0.102, *P* = 0.437) did not explain the source of heterogeneity. Sensitivity analysis showed that none of the studies had an effect on the overall effect size, which suggested that the results of the studies were reliable.

#### 3.7.2. Bacterial positivity

A total of eight studies were enrolled, with seven moderate and one high quality studies. The study revealed that bacterial positivity was a risk factor for malnutrition (OR = 2.08, 95% CI 1.26–3.41, *P* = 0.004), with significant heterogeneity between studies (I^2^ = 79.1%, *P* < 0.001). Meta-regression was not performed as the number of studies was < 10. In addition to the restriction to cross-sectional studies (I^2^ = 81.9%, *P* < 0.001), heterogeneity was not reduced and the combined effect sizes remained significant. Sensitivity analysis showed that the result was stable and reliable.

#### 3.7.3. HIV infection

A total of eight studies were included, all being moderate quality for the studies. The study indicated that HIV infection was not a risk factor for malnutrition (OR = 1.37, 95% CI 0.97–1.93, *P* = 0.070). Heterogeneity between studies was observed (I^2^ = 79.1%, *P* < 0.001). Heterogeneity was still not reduced when limited to cross-sectional studies (I^2^ = 77.0%, *P* = 0.002); however, the pooled results were altered (OR = 1.80, *P* = 0.002). Sensitivity analysis showed that the result was unstable.

#### 3.7.4. Low income

A total of three studies were enrolled and the results showed that low income was a risk factor for malnutrition (OR = 1.44, 95% CI 1.11–1.86, *P* = 0.005), with no heterogeneity between studies (I^2^ = 0.0%, *P* = 0.418). Sensitivity analysis suggested that the result was stable.

#### 3.7.5. Residing in rural areas

A total of four studies were excluded and the results revealed that residing in rural areas was a risk factor for malnutrition (OR = 1.51, 95% CI 1.20–1.89, *P* < 0.001), with no heterogeneity between studies (I^2^ = 0.0%, *P* = 0.690). The sensitivity analysis was stable and reliable.

#### 3.7.6. Smoking

A total of eight studies were included and the results suggested that smoking was a risk factor for malnutrition (OR = 1.40, 95% CI 1.02–1.92, *P* = 0.039) with significant heterogeneity between studies (I^2^ = 76.6%, *P* < 0.001). Heterogeneity was significantly lower when restricted to cross-sectional studies (I^2^ = 27.0%, *P* = 0.241) and high-quality studies (I^2^ = 0.0%, *P* = 0.515); however, the pooled results were not statistically significant. Sensitivity analysis revealed that the result was unstable.

#### 3.7.7. Pulmonary cavity

Six studies were enrolled and the results showed that the pulmonary cavity was a risk factor for malnutrition (OR = 1.59, 95% CI 1.04–2.43, *P* = 0.033) with heterogeneity between studies (I^2^ = 84.4%, *P* < 0.001). When applying restriction to cohort studies, there was no heterogeneity between studies (I^2^ = 0.0%, *P* = 0.417), and the pooled result was still significant (OR = 1.91, *P* < 0.001). However, when applying restriction to high-quality studies, the pooled effect size was not statistically significant (OR = 1.45, *P* = 0.219) and heterogeneity between studies still existed (I^2^ = 90.0%, *P* < 0.001). Sensitivity analyses revealed the result was unstable.

#### 3.7.8. age

A total of three studies were enrolled and the results showed that age ≥65 years was not associated with malnutrition (OR = 1.26, 95% CI 0.54–2.96, *P* = 0.598) with heterogeneity between studies (I^2^ = 70.6%, *P* = 0.033). The sensitivity analysis was unstable.

#### 3.7.9. Family size

Four studies were enrolled for this meta-analysis and the results showed that family size over 4 was a risk factor for malnutrition (OR = 1.33, 95% CI 1.08–1.62, *P* = 0.006) with heterogeneity between studies (I^2^ = 49.8%, *P* = 0.113). The sensitivity analysis was unstable.

#### 3.7.10. Drinking

With eight studies included, the meta-analysis showed that drinking was not a risk factor for malnutrition (OR = 1.17, 95% CI 0.81–1.69, *P* = 0.389), with apparent heterogeneity between studies (I^2^ = 85.4%, *P* < 0.001). When limited to high-quality studies (I^2^ = 0.0%, *P* = 0.512) and cross-sectional studies (I^2^ = 35.9%, *P* = 0.182), heterogeneity was significantly lower and the pooled results were similar to the total effect size. The sensitivity analysis was stable and reliable.

### 3.8. Publication bias

Publication bias assessment was conducted for overall prevalence and the Doi plot revealed major asymmetry (LFK index = 3.87), which suggested that smaller studies may report a higher prevalence for malnutrition (Supplementary Figure 1). Among the risk factors, only the number of studies for “gender” was above 10. The funnel plot showed a slight asymmetry, but Egger's test was not statistically significant (*t* = −0.31, *P* = 0.759), which indicated that there was no publication bias (Supplementary Figure 2).

## 4. Discussion

This study consolidated the current evidence of studies related to the prevalence and risk factors for malnutrition in patients with PTB. A total of 53 studies were included and the pooled prevalence for malnutrition was 48.0% among PTB patients, slightly lower than the 50.8% reported by Wondmieneh et al. (17), which may be related to the small sample size included in his study. The meta-regression revealed that sample size was a source of heterogeneity for overall prevalence with prevalence decreasing with increasing sample size, which was consistent with the Doi plot and suggested that smaller studies may report a higher prevalence for malnutrition. Therefore, a subgroup analysis was carried out and showed a prevalence of 51.5% for the small sample studies, which was similar to the results reported by Wondmieneh et al. (17). However, the reason why the prevalence was higher in the small sample studies may be that most of them included were from hospitalized patients, and studies have demonstrated that patients who were hospitalized tended to have poorer nutritional status (72).

Although the study noted a higher prevalence of PTB among men (73), there was no significant difference in the prevalence of malnutrition between male and female patients with PTB. The meta-analysis showed that male was not associated with malnutrition, which further confirmed that gender was not a risk factor for malnutrition in patients with PTB. Currently, this study showed that the prevalence of severe malnutrition was higher than mild and moderate in patients with PTB, the possible reasons for this are as follows: (1) the studies included were mainly from Africa and Asia, an area with a larger population in rural, lack of medical resources, and poor health awareness, where patients were often not treated adequately and timely due to delayed access to care (4, 74), which may lead to an increased risk for malnutrition and a higher rate for severe malnutrition, and our study also demonstrated that the prevalence for malnutrition was significantly higher in Asia and Africa than America. (2) In addition, a previous study showed a high proportion of drug resistance in Asia and Africa (75), which makes it harder to treat, longer treatment cycles, and poor adherence to treatment among these patients, with a higher risk for severe malnutrition due to the persistence of active PTB (76, 77).

In this study, the prevalence of malnutrition in patients with bacterial positivity was significantly higher than bacteria negative, and bacterial positivity was a risk factor for malnutrition. Walker et al. (78) observed that patients with bacterial positivity had significantly higher levels of inflammatory factors, such as matrix metalloproteinase 1 and matrix metalloproteinase 8, than negative patients, and the stronger the inflammatory response, the more active the catabolism, the greater the energy expenditure, and the greater the risk for malnutrition. Moreover, malnutrition can also lead to delayed sputum conversion (16). Although patients with bacterial positivity were prone to malnutrition and poor outcomes, a study showed that nutritional support not only reduced the risk for malnutrition by suppressing the inflammatory response but also improved the sputum smear or culture conversion rate of PTB (79). Therefore, it was beneficial for the treatment of patients with PTB by nutritional support early.

In our study, the prevalence of malnutrition was significantly higher among patients with pulmonary cavities than those without cavities. A study suggested that patient with a pulmonary cavity on chest radiograph was associated with higher bacterial positivity and a higher bacterial load with thick-walled cavities in patients (80, 81); at the same time, patients with pulmonary cavities always had lower success rates of treatment and longer cycles of treatment (82, 83), which made patients who suffered from PTB with cavities more likely to develop malnutrition. Although this meta-analysis revealed that the pulmonary cavity was a risk factor for malnutrition, sensitivity analysis showed that the results were unstable. Therefore, the conclusions need to be interpreted with caution. One study showed that the prevalence of malnutrition increased with age (84). However, our study concluded the opposite, and age was not a risk factor for malnutrition in patients with PTB. Through the analysis of the included studies, we noted that the same three studies were included both in the older and the younger age groups, and the prevalence of malnutrition was higher in the older than that in the younger group. Because fewer studies were included in the older age group and the number difference between the two groups was large, it could not be assumed that the prevalence of malnutrition was lower in the older age group and the result needed to be reassessed.

This meta-analysis revealed that malnutrition was more prevalent among PTB patients with low income, residing in rural areas, and larger family sizes. According to a meta-analysis, the social economic status has a significant impact on a patient's prognosis for tuberculosis, and low-income patients are more likely to experience treatment failure and multidrug resistance (85). Patients with low income and residing in rural areas had difficulty accessing healthcare and often experienced delayed access, which caused malnutrition due to the persistence of active PTB (69, 86). Whereas, the family size affected the educational status of family members and the distribution of food to a certain extent for less economically developed areas (45, 87), which made patients more vulnerable to malnutrition due to poor health awareness and inadequate intake. This study also showed a significant association between low income and residing in rural areas and malnutrition in PTB, which further confirmed that low income and residing in rural areas were important risk factors for malnutrition. Although the family size was associated with malnutrition in patients with PTB, the results of sensitivity analysis were unstable, so the relationship between them needed to be explored further.

In addition, malnutrition was more prevalent with HIV infection among PTB patients. Patients with HIV infection often had various degrees of immune deficiency and were prone to occurring opportunistic infections, which lead to more energy expenditure and increased nutritional requirements (88, 89). At the same time, people with HIV infection may suffer from oral candidiasis, anorexia, intestinal mucosal damage, and diarrhea, with resulting deficiencies in intake and impairment of absorption (89, 90). Therefore, patients with HIV infection were more likely to be malnourished. However, this study was unclear whether HIV infection was a risk factor for malnutrition in patients with PTB due to the instability of sensitivity analysis, but the outcome suggested that HIV infection tended to increase malnutrition. Furthermore, Montes et al. (10) showed that HIV infection was significantly associated with poor prognosis in patients with PTB, thus it was important to screen for HIV and provide antiviral treatment timely. We also assessed the relationship between smoking and drinking and malnutrition. The meta-analysis showed that smoking increased the risk of malnutrition, but the sensitivity analysis was unstable. In contrast, drinking was not a risk factor for malnutrition; although it was not associated with malnutrition, it was necessary to avoid drinking to prevent exacerbating liver damage during anti-tuberculosis drug treatment (91).

Malnutrition is strongly associated with clinical outcomes in patients with tuberculosis. Several prospective cohort studies have shown that malnutrition is a risk factor for treatment failure, relapse, and death in patients with tuberculosis (92–94). Therefore, it is important to focus on the nutritional status and the risk factors of malnutrition in patients with PTB in clinical management.

### 4.1. Strengths and limitations

There were several advantages to the systematic review and meta-analysis. The first benefit of this study is the consolidated data on the prevalence of malnutrition and risk factors in PTB patients around the world, which will help to improve the management of PTB patients. Second, this meta-analysis displayed a larger sample size, a wider range of research, and more reliable results compared to earlier research. Finally, we searched multiple databases to minimalize the omission of relevant studies. However, there were some limitations to our study. First, some indicators were characterized by high heterogeneity between studies, a small number of studies, and an unstable sensitivity analysis, which suggests that more prospective cohort studies are needed to further evaluate the relationship among HIV infection, age, family size, smoking, pulmonary cavity, and malnutrition in the future, so the results should be explained with caution. Second, as the majority of the studies were retrospective observational studies with an unclear sequence of exposure and outcome, the results should be treated with caution. Third, only one study was from Europe, which made it impossible to provide an accurate assessment of the prevalence of malnutrition among PTB patients in Europe. Finally, we only included studies published in English, which may cause bias in the results due to missing important studies published in other languages.

## 5. Conclusion

In summary, this meta-analysis revealed that the prevalence for malnutrition was quite prevalent in patients with PTB, and bacterial positivity, low income, and residing in rural areas were significantly associated with malnutrition in patients with PTB. Therefore, we urge clinicians and TB patients to pay attention to the screening and prevention of malnutrition. Early identification of malnutrition and its risk factors in patients with PTB facilitates timely nutritional support treatment for patients with PTB, which will prevent further development and deterioration of PTB.

## Data availability statement

The original contributions presented in the study are included in the article/Supplementary material, further inquiries can be directed to the corresponding author.

## Author contributions

Study design: AL and N-nL. Data search and extraction: AL and S-yY. Data analysis and writing: AL, S-yY, Q-gL, and J-xL. Manuscript revision: X-yY and N-nL. All authors contributed to the article and approved the submitted version.
