# Supplementary material for: Prevalence and risk factors of malnutrition in patients with pulmonary tuberculosis: a systematic review and meta-analysis
Source: Front Med (Lausanne). 2023 Aug 10;10:1173619. doi: 10.3389/fmed.2023.1173619 (PMC10448260; doi:10.3389/fmed.2023.1173619)

Supplemental Table 1. The search strategy for each database.

| Databases | Search strategy |
| --- | --- |
| PubMed | #1 "Tuberculosis"[Mesh] OR " Tuberculosis, Pulmonary"[Mesh]  #2 ((((((((((Tuberculoses[Title/Abstract]) OR (Kochs Disease[Title/Abstract])) OR (Koch's Disease[Title/Abstract])) OR (Koch Disease[Title/Abstract])) OR (Mycobacterium tuberculosis Infection[Title/Abstract])) OR (Infection, Mycobacterium tuberculosis[Title/Abstract])) OR (Infections, Mycobacterium tuberculosis[Title/Abstract])) OR (Mycobacterium tuberculosis Infections[Title/Abstract])) OR (Tuberculoses, Pulmonary[Title/Abstract])) OR (Pulmonary Tuberculoses[Title/Abstract])) OR (Pulmonary Tuberculosis[Title/Abstract])  #3 #1 OR #2  #4 "Malnutrition"[Mesh]  #5 (((((((((((((Nutritional Deficiency[Title/Abstract]) OR (Nutritional Deficiencies[Title/Abstract])) OR (Undernutrition[Title/Abstract])) OR (Malnourishment[Title/Abstract])) OR (Malnourishments[Title/Abstract])) OR (weight loss[Title/Abstract])) OR (nutrient loss[Title/Abstract])) OR (nutritional status[Title/Abstract])) OR (Anorexia[Title/Abstract])) OR (thin[Title/Abstract])) OR (Cachexia[Title/Abstract])) OR (nutritional imbalance[Title/Abstract])) OR (Nutrition[Title/Abstract])) OR (nutritional level[Title/Abstract])  #6 #4 OR #5  #7 "Prevalence"[Mesh] OR "Epidemiology"[Mesh] OR "Incidence"[Mesh] OR "Risk Factors"[Mesh]  #8 (((((((Prevalences[Title/Abstract]) OR (Incidences[Title/Abstract])) OR (Attack Rate[Title/Abstract])) OR (Attack Rates[Title/Abstract])) OR (Risk Factor[Title/Abstract])) OR (risk[Title/Abstract])) OR (factor[Title/Abstract])) OR (factors[Title/Abstract])  #9 #7 OR #8  #10 #3 AND #6 AND #9 |
| Embase | #1 'tuberculosis'/exp  #2 'tuberculosis, pulmonary'/exp  #3 'tuberculoses':ab,ti OR 'mycobacterium tuberculosis infection':ab,ti OR 'infection, mycobacterium tuberculosis':ab,ti OR 'infections, mycobacterium tuberculosis':ab,ti OR 'mycobacterium tuberculosis infections':ab,ti OR 'tuberculoses, pulmonary':ab,ti OR 'pulmonary tuberculoses':ab,ti OR 'pulmonary tuberculosis':ab,ti  #4 #1 OR #2 OR #3  #5 'malnutrition'/exp  #6 'nutritional deficiency':ab,ti OR 'nutritional deficiencies':ab,ti OR 'undernutrition':ab,ti OR 'malnourishment':ab,ti OR 'malnourishments':ab,ti OR 'weight loss':ab,ti OR 'nutrient loss':ab,ti OR 'anorexia':ab,ti OR 'thin':ab,ti OR 'nutritional status':ab,ti OR 'nutritional imbalance':ab,ti OR 'cachexia':ab,ti OR 'nutrition':ab,ti OR 'nutritional level':ab,ti  #7 #5 OR #6  #8 'prevalence'/exp  #9 'epidemiology'/exp  #10 'incidence'/exp  #11 'risk factors'/exp  #12 'prevalences':ab,ti OR 'incidences':ab,ti OR 'attack rate':ab,ti OR 'attack rates':ab,ti OR 'risk factor':ab,ti OR 'risk':ab,ti OR 'factor':ab,ti OR 'factors':ab,ti  #13 #8 OR #9 OR #10 OR #11 OR #12  #14 #4 AND #7 AND #13 |
| Web of science | #1 TS=(tuberculosis or Tuberculosis, Pulmonary or Tuberculoses or Mycobacterium tuberculosis Infection or Infection, Mycobacterium tuberculosis or Infections, Mycobacterium tuberculosis or Mycobacterium tuberculosis Infections or Tuberculoses, Pulmonary or Pulmonary Tuberculoses or Pulmonary Tuberculosis)  #2 TS=(malnutrition or Nutritional Deficiency or Nutritional Deficiencies or Undernutrition or Malnourishment or Malnourishments or weight loss or nutrient loss or nutritional status or Anorexia or thin or nutritional imbalance or Cachexia or Nutrition or nutritional level)  #3 TS=( Prevalence or Epidemiology or Incidence or Risk Factors or Prevalences or Incidences or Attack Rate or Attack Rates or Risk Factor or risk or factor or factors)  #3 #1 AND #2 AND #3 |
| The Cochrane Library | #1 [Tuberculosis] explode all trees  #2 [Tuberculosis, Pulmonary] explode all trees  #3 (Tuberculoses):ti,ab,kw OR (Mycobacterium tuberculosis Infection):ti,ab,kw OR (Infection, Mycobacterium tuberculosis):ti,ab,kw OR (Infections, Mycobacterium tuberculosis):ti,ab,kw OR (Mycobacterium tuberculosis Infections):ti,ab,kw OR (Tuberculoses, Pulmonary):ti,ab,kw OR (Pulmonary Tuberculoses):ti,ab,kw OR (Pulmonary Tuberculosis):ti,ab,kw  #4 #1 OR #2 OR #3  #5 [Malnutrition] explode all trees  #6 (Nutritional Deficiency):ti,ab,kw OR (Nutritional Deficiencies):ti,ab,kw OR (Undernutrition):ti,ab,kw OR (Malnourishment):ti,ab,kw OR (Malnourishments):ti,ab,kw OR (weight loss):ti,ab,kw OR (nutrient loss):ti,ab,kw OR (nutritional status):ti,ab,kw OR (Anorexia):ti,ab,kw OR (thin):ti,ab,kw OR (nutritional status):ti,ab,kw OR (nutritional imbalance):ti,ab,kw OR (Cachexia):ti,ab,kw OR (Nutrition):ti,ab,kw OR (nutritional level):ti,ab,kw  #7 #5 OR #6  #8 [Epidemiology] explode all trees  #9 [Incidence] explode all trees  #10 [Prevalence] explode all trees  #11 [Risk Factors] explode all trees  #12 (Prevalences):ti,ab,kw OR (Incidences):ti,ab,kw OR (Attack Rate):ti,ab,kw OR (Attack Rates):ti,ab,kw OR (Risk Factor):ti,ab,kw OR (risk):ti,ab,kw OR (factor):ti,ab,kw OR (factors):ti,ab,kw  #13 #8 OR #9 OR #10 OR #11 OR #12  #14 #4 AND #7 AND #13 |

Supplemental Table 2. Agency for Healthcare Research and Quality

| Study’s  first author | Q1 | Q2 | Q3 | Q4 | Q5 | Q6 | Q7 | Q8 | Q9 | Q10 | Q11 | Score |
| --- | --- | --- | --- | --- | --- | --- | --- | --- | --- | --- | --- | --- |
| Baluku | 1 | 1 | 1 | 1 | 0 | 1 | 1 | 1 | 0 | 1 | 0 | 8 |
| Zafar | 1 | 1 | 1 | 1 | 0 | 1 | 0 | 1 | 0 | 1 | 1 | 8 |
| Tao | 1 | 1 | 1 | 1 | 0 | 1 | 0 | 1 | 0 | 1 | 0 | 7 |
| Li | 1 | 1 | 1 | 1 | 0 | 1 | 0 | 1 | 0 | 0 | 0 | 6 |
| Hussien | 1 | 1 | 1 | 1 | 0 | 1 | 0 | 1 | 0 | 1 | 0 | 7 |
| Song | 1 | 1 | 1 | 1 | 0 | 1 | 1 | 1 | 0 | 1 | 0 | 8 |
| Kassa | 1 | 0 | 1 | 1 | 0 | 1 | 0 | 1 | 0 | 1 | 0 | 6 |
| Montes | 1 | 1 | 1 | 1 | 0 | 1 | 0 | 1 | 0 | 1 | 0 | 8 |
| Kitonsa | 1 | 0 | 1 | 1 | 0 | 1 | 0 | 1 | 0 | 1 | 0 | 6 |
| Shimouchi | 1 | 1 | 1 | 1 | 0 | 1 | 1 | 0 | 0 | 1 | 0 | 7 |
| Musuenge | 1 | 1 | 0 | 1 | 0 | 1 | 1 | 1 | 0 | 0 | 0 | 6 |
| Ko | 1 | 1 | 1 | 1 | 0 | 1 | 1 | 0 | 0 | 0 | 0 | 6 |
| White | 1 | 1 | 1 | 1 | 0 | 1 | 0 | 1 | 0 | 0 | 0 | 6 |
| Campos | 1 | 1 | 0 | 0 | 0 | 1 | 0 | 1 | 0 | 0 | 0 | 4 |
| Kubiak | 1 | 1 | 1 | 1 | 0 | 1 | 0 | 1 | 0 | 1 | 0 | 7 |
| Rashak | 1 | 0 | 1 | 1 | 0 | 1 | 0 | 1 | 0 | 1 | 0 | 6 |
| Hoyt | 1 | 1 | 1 | 1 | 0 | 1 | 0 | 1 | 0 | 0 | 0 | 6 |
| Hussien | 1 | 0 | 1 | 1 | 0 | 1 | 0 | 1 | 0 | 0 | 0 | 5 |
| Feleke | 1 | 1 | 1 | 1 | 0 | 1 | 0 | 1 | 0 | 1 | 0 | 7 |
| Lazzari | 1 | 1 | 0 | 1 | 0 | 1 | 0 | 1 | 0 | 0 | 0 | 5 |
| Patsche | 1 | 1 | 1 | 1 | 0 | 1 | 0 | 1 | 0 | 1 | 0 | 7 |
| Hochberg | 1 | 1 | 1 | 0 | 0 | 1 | 0 | 1 | 0 | 1 | 0 | 6 |
| Lawson | 1 | 1 | 1 | 1 | 0 | 1 | 0 | 1 | 0 | 1 | 0 | 7 |
| Lettow | 1 | 1 | 1 | 1 | 0 | 1 | 0 | 0 | 0 | 1 | 0 | 6 |
| Madebo | 1 | 1 | 1 | 1 | 0 | 1 | 0 | 1 | 0 | 1 | 0 | 7 |
| Muchsin | 1 | 0 | 0 | 0 | 0 | 1 | 0 | 1 | 0 | 0 | 0 | 3 |
| Swaminathan | 1 | 1 | 1 | 1 | 0 | 1 | 0 | 1 | 0 | 1 | 0 | 7 |
| Sari | 1 | 1 | 1 | 1 | 0 | 1 | 0 | 1 | 0 | 0 | 0 | 6 |
| Salam | 1 | 1 | 0 | 0 | 0 | 1 | 0 | 1 | 0 | 0 | 0 | 4 |
| PrayGod | 1 | 1 | 1 | 1 | 0 | 1 | 0 | 1 | 0 | 0 | 0 | 6 |
| Piva | 1 | 0 | 1 | 1 | 0 | 1 | 0 | 1 | 0 | 1 | 0 | 6 |
| Dodor | 1 | 1 | 1 | 1 | 0 | 1 | 0 | 1 | 0 | 1 | 0 | 7 |
| Lin | 1 | 1 | 1 | 1 | 0 | 1 | 0 | 1 | 0 | 0 | 0 | 6 |
| Pakasi | 1 | 1 | 0 | 1 | 0 | 1 | 0 | 0 | 0 | 0 | 0 | 4 |

Notes: “1” represents Yes; “0” represents No or Unclear

Q1: Define the source of information (survey, record review)

Q2: List the inclusion and exclusion criteria for exposed and unexposed subjects (cases and controls) or refer to previous publications

Q3: Indicate time period used for identifying patients

Q4: Indicate whether or not subjects were consecutive if not population-based

Q5: Indicate if evaluators of subjective components of study were masked to other aspects of the status of the participants

Q6: Describe any assessments undertaken for quality assurance purposes (e.g., test/retest of primary outcome measurements)

Q7: Explain any patient exclusion from analysis

Q8: Describe how confounder was assessed and/or controlled

Q9: If applicable, explain how missing data were handled in the analysis

Q10: Summarize patient response rates and completeness of data collection

Q11: Clarify what follow-up, if any, was expected and the percentage of patients for which incomplete data or follow-up was obtained

Supplemental Table 3. Newcastle-Ottawa Quality Assessment Scale

| Study’s  first author | Selection  (0–4 stars) | | | | Comparability  (0–2 stars) | Outcome  (0–3stars) | | | Score |
| --- | --- | --- | --- | --- | --- | --- | --- | --- | --- |
|  | Q1 | Q2 | Q3 | Q4 | Q5 | Q6 | Q7 | Q8 |  |
| Soeroto | - | * | * | * | ** | * | * | - | 7 |
| Magassouba | - | * | * | * | - | * | * | * | 6 |
| Asemahagn | - | * | * | * | - | * | * | - | 5 |
| Sahile | * | * | * | * | - | * | * | * | 7 |
| Singla | - | * | * | * | - | * | - | * | 5 |
| Baluku | * | * | * | - | * | * | * | - | 6 |
| Kornfeld | * | * | * | * | - | * | * | - | 6 |
| Yen | * | * | * | * | - | * | * | * | 7 |
| Park | - | * | * | * | ** | * | * | * | 8 |
| Chung | - | * | * | * | - | * | * | * | 6 |
| Putri | - | * | * | * | * | * | * | * | 7 |
| Bhargava | * | * | * | * | - | * | * | * | 7 |
| Mupere | * | * | * | * | - | * | * | - | 6 |
| Podewils | * | * | * | * | ** | * | * | - | 8 |
| Kawai | * | * | * | - | - | * | * | * | 6 |
| Kennedy | * | * | * | - | - | * | * | - | 5 |
| Nandasena | * | * | * | * | - | * | * | - | 6 |
| Frediani | * | * | * | - | - | * | * | - | 5 |
| Gler | - | * | * | - | * | * | * | - | 5 |

Notes:“*” represents one point

Q1: Representativeness of the exposed cohort

Q2: Selection of the non-exposed cohort

Q3: Ascertainment of exposure

Q4: Demonstration that outcome of interest was not present at the start of the study

Q5: Comparability of cohorts on the basis of the design or analysis

Q6: Assessment of outcome

Q7: Was followed up long enough for outcomes to occur

Q8: Adequacy of follow-up of cohorts

Supplemental Figure 1. Publication bias of overall prevalence for malnutrition in patients with PTB.


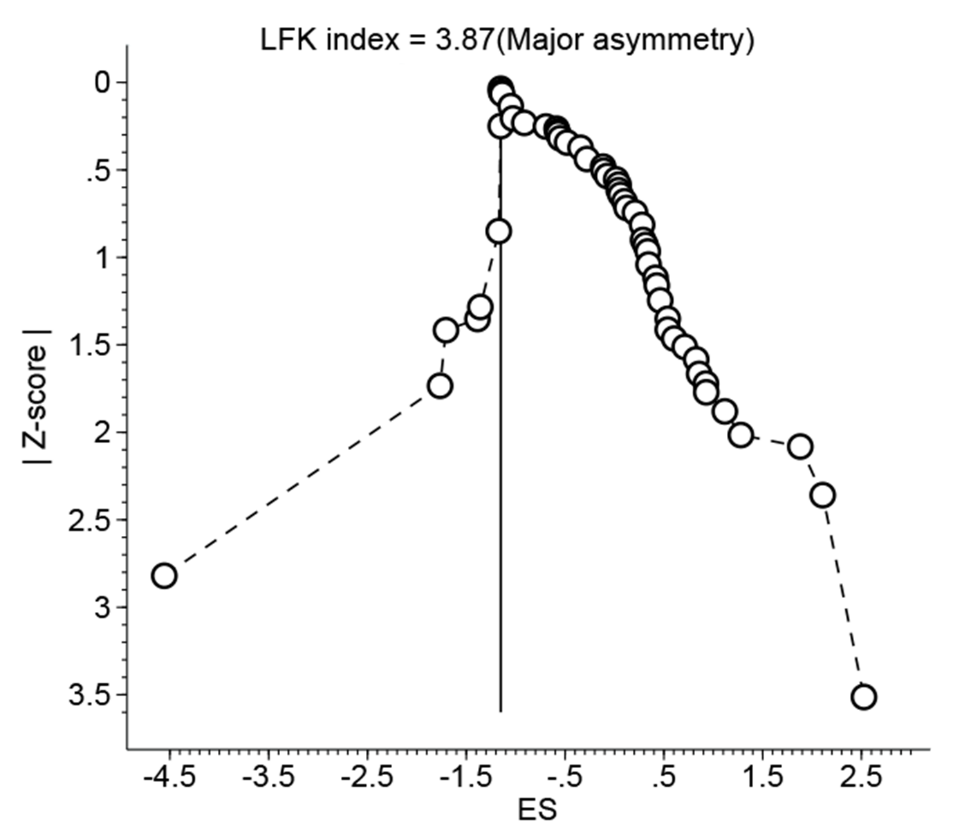


Supplemental Figure 2. Funnel plot of male and malnutrition.


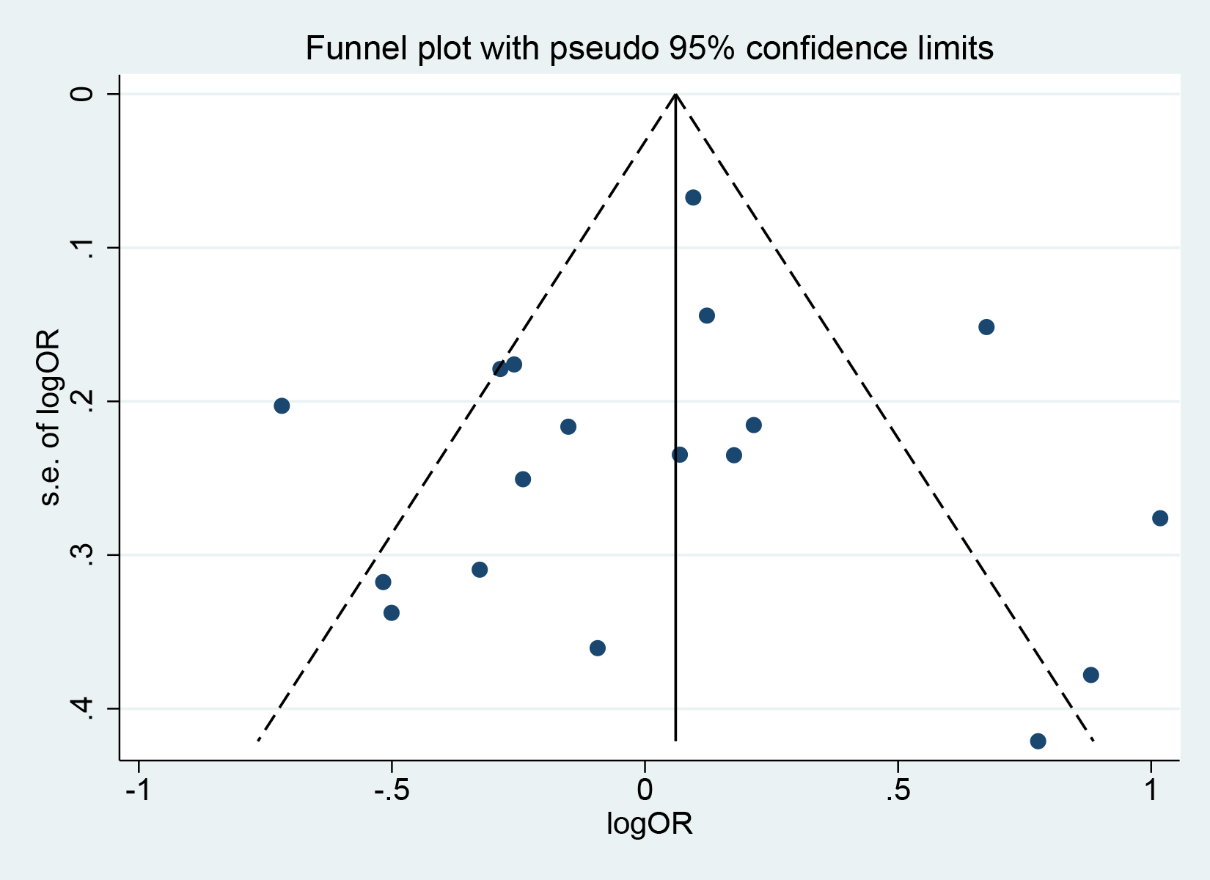

Supplement: Supplementary file 1 [file Data_Sheet_1.docx]
